# Exploration of the Antimicrobial Synergy between Selected Natural Substances on Streptococcus mutans to Identify Candidates for the Control of Dental Caries

**DOI:** 10.1128/spectrum.02357-21

**Published:** 2022-04-12

**Authors:** Alisha Prince, Soumya Roy, David McDonald

**Affiliations:** a Wichita State University, Wichita, Kansas, USA; b B-Gyan LLC., Plainville, Massachusetts, USA; Ohio State University

**Keywords:** Streptococcus mutans, cranberry extracts, proanthocyanidins, manuka honey, methylglyoxal, antimicrobial combinations, antimicrobial synergy

## Abstract

Dental caries is caused by the buildup of acidic end products that result from the metabolism of dental plaque microbes. Natural products that are widely available could be used as an alternative or adjunctive anti-caries therapy. Sometimes, when two products are used together, they yield a more powerful antimicrobial effect than the anticipated additive effect. These synergistic combinations are often better treatment options because individual agents may not have sufficient antimicrobial action to be effective when used alone. Cranberries contain phenolic compounds like proanthocyanidins (PAC) that disrupt biofilm formation. Manuka honey has high concentrations of the agent methylglyoxal (MGO), which is cariostatic. Because these agents have varied modes of antimicrobial action, they show potential for possible synergistic effects when paired. Various cranberry extracts were tested pairwise with manuka honey or MGO by well-diffusion assays and 96-well checkerboard assays in the presence of Streptococcus mutans to test for synergy. Synergy was demonstrated in cranberry extracts Type R and RE when paired with manuka honey and MGO. The synergistic combinations found in this research thus can be considered candidates for the formulation of a dentifrice that could be used to inhibit the formation of dental plaque and thereby avoid the development of caries.

**IMPORTANCE** The emergence of bacteria resistant to antimicrobial agents has led to a shortage of options when choosing effective treatment agents. Further, some antibiotics used at therapeutic doses can produce undesired side effects. An alternative to traditional antibiotics, natural antimicrobial agents can be used in combination to obtain synergistic outcomes without subjecting the patient to toxic or irritating doses of individual agents. Streptococcus mutans growth and biofilm formation are major contributors to the formation of dental caries. In this study, a synergistic combination of Manuka honey and cranberry extracts gives evidence that it can be used as an alternative or adjunctive anti-caries therapy.

## INTRODUCTION

As early as 1924, it was discovered that the bacterium Streptococcus mutans contributes to the association of dental plaque and tooth decay (1). *In vivo* studies with germ-free rats corroborated these findings, proving that tooth decay and gum disease are caused by bacteria (2). S. mutans is a facultative anaerobic Gram-positive coccus. The virulence factors that contribute to its cariogenicity are (i) adhesion to tooth surfaces, (ii) acidogenicity through glycolysis, and (iii) acid tolerance (3). S. mutans encodes glucosyltransferases that can convert the dietary starches to extracellular polymers like glucan that can bind to the dental pellicle, which then allows for the attachment of other bacteria like Streptococcus sanguis, Streptococcus oralis, and *Lactobacillus* sp. (4). The fermentation of simple sugars by this bacterial consortium produces organic acids that can with time erode the enamel surface of the tooth and cause dental caries.

Many studies have been performed to test the activity of natural products like green tea, cacao bean, cranberry, cinnamon, garlic, honey, etc. against S. mutans (5–10). Extracts from these plant materials show significant levels of antimicrobial activity. Further, because they are derived from plant materials that have been consumed by humans for many years, concerns about toxicity or irritation are typically much reduced compared with newly synthesized agents. Indeed, they may even have a flavor profile that is pleasing to the human palate. The hope is that these extracts can be used as alternative or adjunctive anti-caries agents.

Sometimes, when two products are used together, they yield a more powerful antimicrobial effect than the anticipated additive effect. These synergistic combinations are often better treatment options because individual agents may not have sufficient antimicrobial action to be effective when used alone or may require sufficiently high concentrations that they become toxic or irritating (11). Furthermore, exposure to dual agents greatly reduces the likelihood of the development of resistance by the target bacteria. Finally, it is also important to note that some natural substances; for example, in this study cranberry and honey, have pleasant flavor profiles, which would likely lead to increased patient compliance to a regimen that includes a dentifrice composed of these natural substances.

## RESULTS

**FIG 1:**
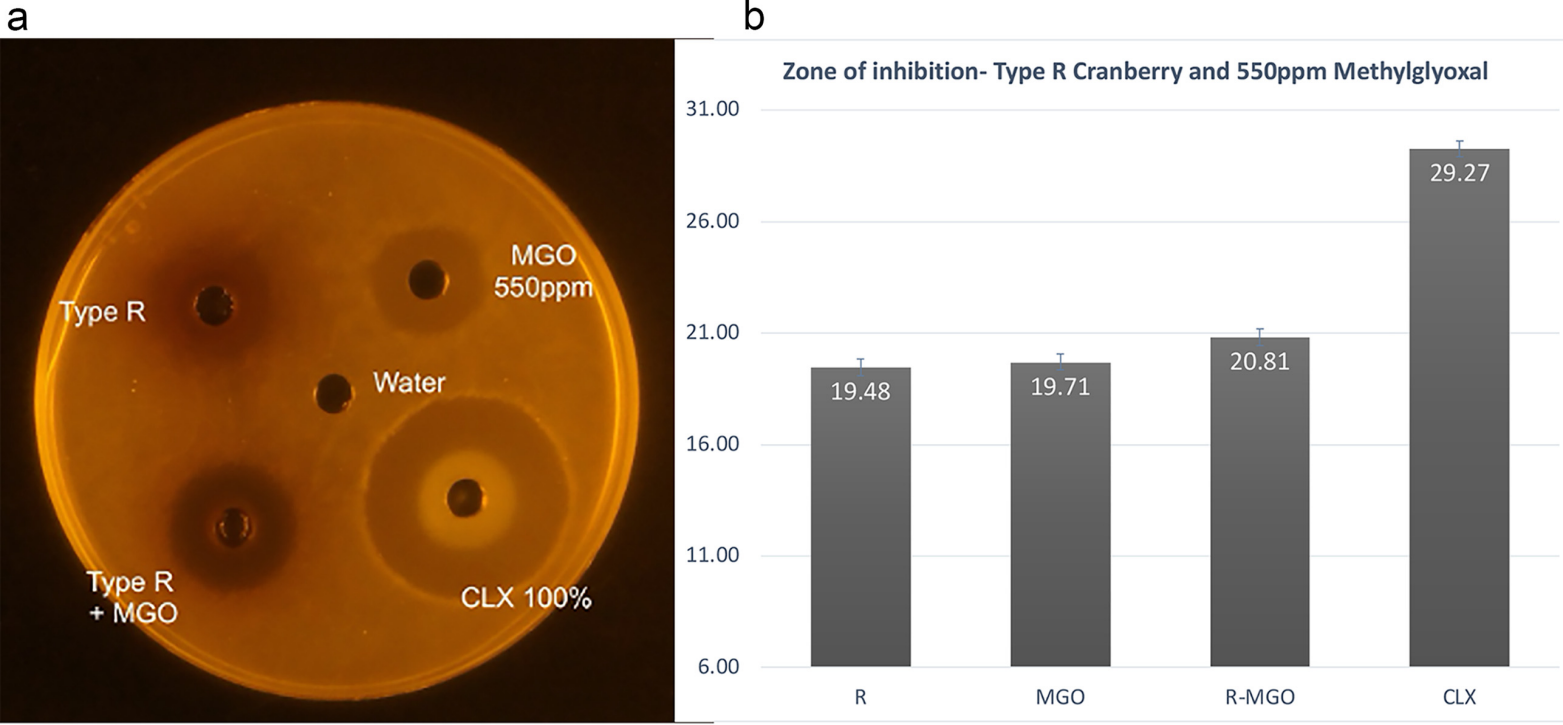
Well diffusion assay: cranberry Type R and MGO (a) Well diffusion assay with each well containing Type R cranberry extract, 550 ppm MGO, Type R-MGO combination and a control chlorhexidine. (b) Bar graph with data collected from 20 petri plates showing the diameter of zones of inhibition around each well. The diameter of the cork borer used to make the wells was 6 mm.

To test if compounds would show synergism when mixed, we set up an agar well diffusion assay (Fig. 1a). The antimicrobial potency was measured by the diameter of the zones of growth inhibition. Of the five cranberry extracts tested, two extracts (Type R and RE) when paired with manuka honey, showed stronger antimicrobial action in the well-diffusion assay. The zones of inhibition around the cranberry-manuka honey combination wells were larger than those around the cranberry and manuka honey wells, respectively. This difference was statistically significant (*P* < 0.0001) and repeatable, with consistent results when the experiments were performed in triplicate (Fig. 1b). Cranberry extracts Type R and RE, when paired with 550 ppm MGO showed significantly (*P* < 0.0001) larger zones of inhibition when used in combination with MGO, than when used alone.

**FIG 2:**
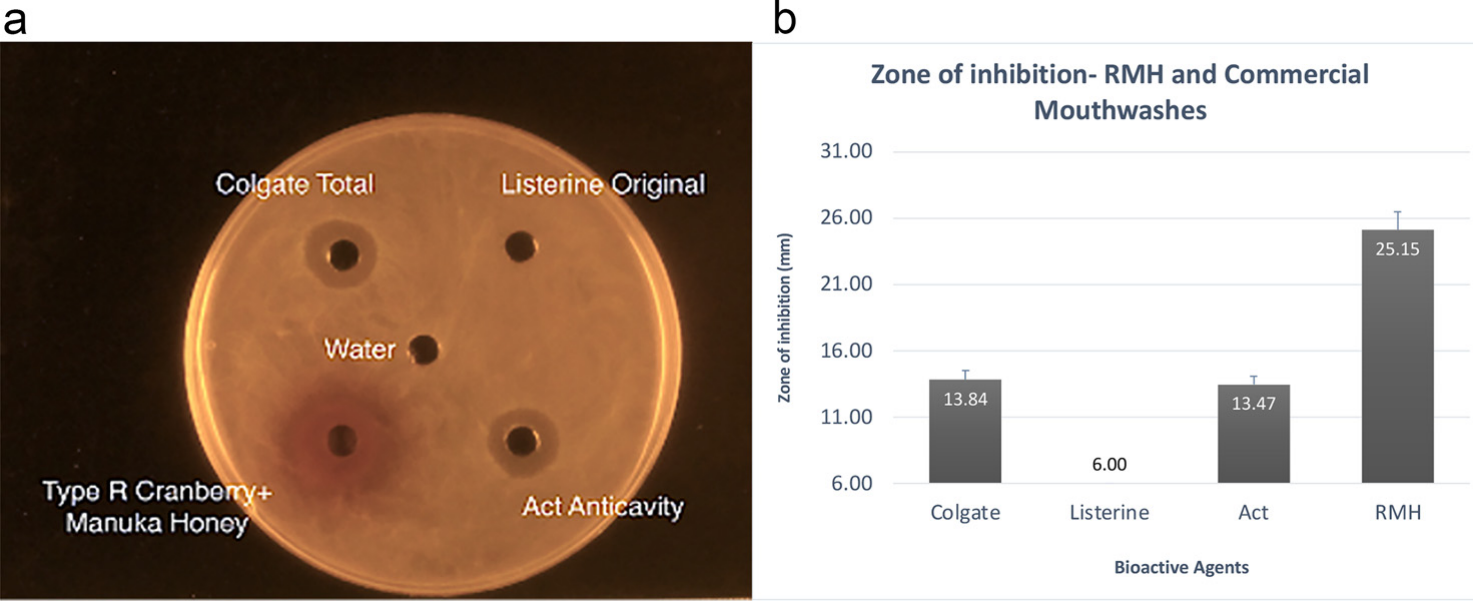
Well diffusion assay with cranberry Type R-manuka honey combination, and commercial mouthwashes. (a) Well diffusion assay with each well containing Colgate Total, Listerine Original, ACT anticavity mouthwashes and Type R-manuka honey combination. (b) Zones of inhibition around wells containing Type R-manuka honey combination, and commercial mouthwashes in a well diffusion assay with S. mutans from 20 petri plates. The cork borer diameter used to prepare the wells was 6 mm.

The Type R – manuka honey combination was subjected to a well diffusion assay along with commercially available mouthwash (Fig. 2a). The zones of inhibition around the Type R-manuka honey combination were much larger than those around the mouthwashes. The Type R and manuka honey combination had statistically larger (*P* < 0.0001) zones of inhibition than the mouthwashes. Colgate Total and ACT mouthwashes had statistically similar zones of inhibition. Listerine had no zones of inhibition in this well diffusion assay (Fig. 2b).

**FIG 3:**
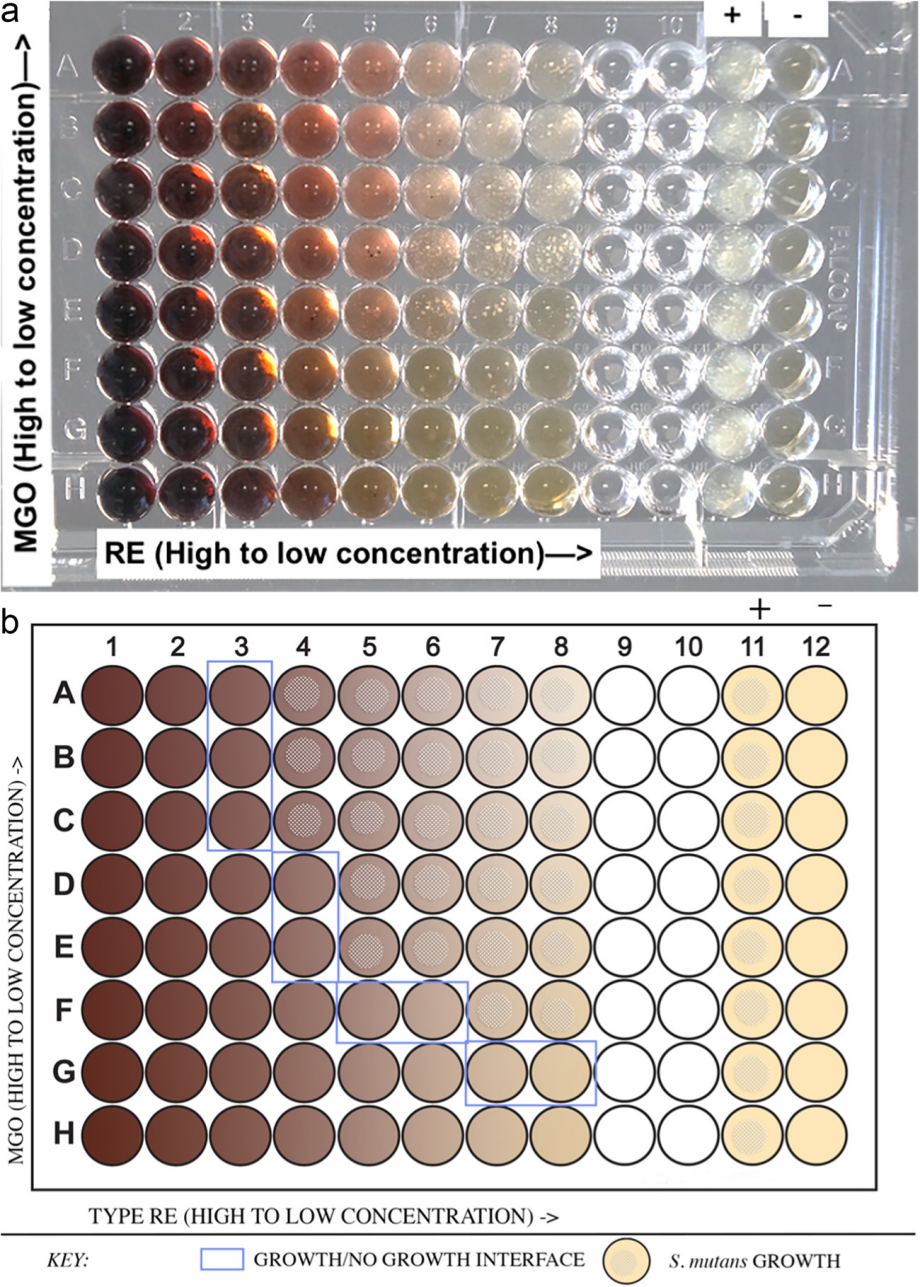
Checkerboard Assay setup. (a) Cranberry extract RE in serial dilutions along the columns 1 to 8. 550 ppm MGO in serial dilutions along the rows A to H. Column 11 is a positive growth control with BHI broth and *S.mutans*. Column 12 is a negative growth control with only BHI broth. (b) Illustration of the same 96-well plate, showing the growth/no growth interface, which is the first well without growth in every row and column.

As some natural extracts tend to polymerize and have trouble diffusing into the agar in well diffusion assay, a 96-well plate checkerboard assay was used to test synergy (Fig. 3a and b). Cranberry extracts Type R, RE and SWPE, in their original concentrations, when combined with MGO, showed synergy (Table 1). The mean FIC index for cranberry extracts Type R, RE and SWPE, when combined with MGO were 0.6, 0.5 and 0.8, respectively, which were less than 1, indicating synergy (12). This study was replicated and showed similar data. The MIC of 550 ppm concentration MGO was 0.5. Type RE and SWPE had MICs of 1. As cranberry extracts had an MIC of 1, serial dilutions of intermediate concentrations (0.75, 0.38, 0.19, etc.) were added to checkerboard assays. This gave an MIC of 0.75 for RE and 0.75 for SWPE, indicating that the MIC of RE and SWPE were between 0.75 and 1. MIC for Type R was 0.5. The calculated mean FIC index for this study remained at 0.5 indicating synergy. The antimicrobial combinations effects of all five cranberry extracts and manuka honey or MGO combinations are summarized in Table 2.

**TABLE 1:**
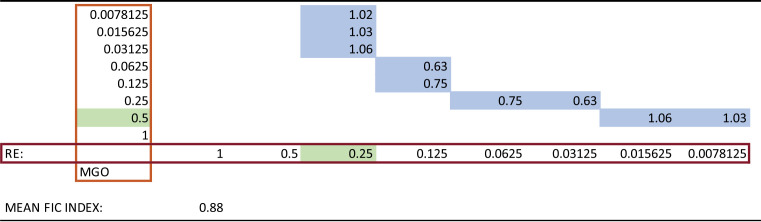
Mean FIC index of plate shown in Fig. 3 MGO in serial dilutions along the rows. MGO in serial dilution along the columns^*a*^

a Blue indicates FICs of each well along the growth/no growth interface. Green indicates MICs of RE and MGO. The mean FIC index is the average of all FICs along the growth/no growth interface.

**TABLE 2:** Antimicrobial combination effects in well diffusion and checkerboard assay

Product a	Product B	Well diffusion assay	Checkerboard assay
Type R	Manuka honey	Synergy	Synergy
	MGO	Synergy	Synergy
RE	Manuka honey	Synergy	Synergy
	MGO	Synergy	Synergy
SWP	Manuka honey	Additive	Additive
	MGO	Additive	Synergy
SWPE	Manuka honey	Additive	Synergy
	MGO	Additive	Synergy
SWF	Manuka honey	Additive	Additive
	MGO	Additive	Additive

## DISCUSSION

Synergy is demonstrated when two or more separate products have a combined effect that is greater than the sum of the individual products. When the combined effect is equal to the sum of individual products, they are additive. If products, when combined, are less effective than when used alone, the phenomenon is antagonistic. Cranberry extracts R and RE when paired with Manuka honey and MGO, respectively, showed synergy in both the well-diffusion assay and the checkerboard assay. This may be the consequence of their varied modes of action being complementary against S. mutans. Perhaps future studies will reveal how they can be used in combination with other antimicrobial agents to achieve a level of antimicrobial activity useful for dental applications.

It is well-known that dietary sugars contribute to the progression of dental caries and oral biofilm formation (13). Manuka honey, like most honeys has a high sugar content. Although manuka honey was synergistic with cranberry in this study, by substituting Manuka honey with one of its main bioactive agents, MGO, we can avoid sugars, if this is to be used orally as a dentifrice.

One of the main drawbacks of the well diffusion assay is that some natural products with larger molecule sizes could not diffuse into the BHI agar leading to false negative results. The color of cranberry extracts surrounding the well, indicated the level of diffusion into the agar. Cranberry extracts have proanthocyanidins (PAC) that polymerize over time while maintaining their antimicrobial properties. But the polymerized components could not diffuse into the agar in the well diffusion assay. To test synergy between these products, the checkerboard assay was used. Cranberry PAC that polymerizes to form larger molecules were more bioactive in the checkerboard assay than the well diffusion assay and were antimicrobial even after polymerization, as evidenced by the inhibition of bacterial growth. Therefore, while testing natural products it is important to keep in mind the structure of the bioactive molecules with respect to the assay that is being used to test it.

The checkerboard assay has many advantages. The liquid broth medium avoids the agar diffusion problem faced by products with larger molecule sizes. We can simultaneously test combinations of various concentrations of both the test substances. The MIC and FIC for each antimicrobial combination can be determined in a single plate.

Some research (14) suggest that synergy is present when the mean FIC index is ≤ 0.5, additive/indifference is present when FIC index is between 1 and 4, and antagonism is more than 4. The 0.5 conservative number will account for random effects in a microdilution checkerboard assay. But as Meletiadis et al. describe, a 0.5 FIC is not a natural cut off, making it difficult to classify FICs between 0.5 and 1 (12, 15). Run-to-run values do not vary to a point where synergy is demonstrated in one run and antagonism is seen in another run (16, 17). It seems better to go with the values described above.

While these results show potential for some synergistic pairs, more research is required before these extracts can be assessed for use as a dentifrice for human patients. The oral biofilm is complex, with multiple species of bacteria embedded in a polymeric matrix. The effect of these natural agents on the biofilm should also be studied. A crystal violet biofilm assay can be used to determine the antimicrobial activity of synergistic pairs against the formation of new biofilm. A potential difficulty for using this method to test cranberries or similar compounds is that the agents have absorption wavelengths close to that of crystal violet and that may lead to difficulties while using a spectrophotometric plate reader. Also, randomized control studies could be performed on small groups of individuals to check the effectiveness of synergistic pairs *in vivo*. The compounds could be used as a mouthwash or incorporated in toothpastes and tested over time to detect the effects on dental plaque and calculus formation. This would also give more data regarding the effects of saliva on the synergistic combinations. By growing periodontal bacteria anaerobically, and subjecting them to well diffusion and checkerboard assays, we may also find synergistic compounds that can treat gum disease. Other plant-derived antimicrobial agents can be tested in both the well diffusion assay and the checkerboard assay. By identifying natural extracts that have proven antimicrobial activity *in vitro* or *in vivo* and combining them with other agents, we can test for synergy. It is important to keep in mind that the likelihood of detecting synergy is higher if the two agents being combined have different antimicrobial mechanisms. If they have similar modes of action, they tend to act in an additive manner and may even be antagonistic.

With the invention of newer technologies, it is crucial to find the right tools that can further the study of antimicrobial synergy and use natural products more effectively to fight tooth decay.

## MATERIALS AND METHODS

All cranberry extracts were provided by Ocean Spray Cranberries Inc. The cranberry extracts were labeled by the manufacturers as “SWP” (subcritical water extract of presscake), “SWF” (subcritical water extract of fruit), “SWPE” (subcritical water extract of presscake with tannase), “Type R” (resin extract) and “RE” (resin extract with tannase). The extracts vary based on their composition and methods of extraction (18). For this experiment, we purchased manuka honey (Manuka Health, New Zealand) containing 550+ ppm MGO. Methylglyoxal (Sigma, USA) was added to distilled water to prepare a 550 ppm solution of methylglyoxal, equivalent to the label-indicated content in the manuka honey. Commercially available mouthwashes Colgate Total, Listerine, and ACT were also used. To prepare a mix of extracts, 750 μL of one extract was added to 750 μL of another extract in a sterile Eppendorf tube and mixed well. Distilled water was used as a negative control. Chlorhexidine (2% vol/vol chlorhexidine gluconate) was used as a positive control.

Brain Heart Infusion (BHI) agar plates were used to culture S. mutans (ATCC 25175) by incubating streak plates in a 5% carbon dioxide (CO_2_) incubator at 37°C overnight. To standardize the bacterial suspension used in each assay, a 0.5 McFarland standard was prepared (19).

### Agar well diffusion assay.

100 μL of S. mutans at a turbidity of 0.5 McFarland standard were added to each test tube with 25 mL BHI agar at 55°C and poured into 20 petri plates. The plates were allowed to sit at room temperature for 2 h. Using a sterile cork-borer of 0.6 mm diameter, five wells were punched in the BHI agar of every petri dish. A template was used to ensure that the wells were equidistant from each other. In each petri dish, 95 μL of cranberry extract SWP, manuka honey, SWP-manuka honey mixture, water and chlorhexidine were pipetted into the wells. After allowing them to diffuse for 30 min, they were placed in a CO_2_ incubator at 37°C overnight (Fig. 1a). This method was repeated with extracts SWF, SWPE, Type R, and RE, respectively, combined with manuka honey. All five cranberry extracts were then combined with MGO and tested. To compare the efficacy of the most antimicrobial combination with commercial mouthwash, 95 μL of the cranberry Type R and manuka honey mixture was pipetted in one well and 95 μL of Colgate Total, Listerine and ACT mouthwashes were pipetted into the three other outer wells, respectively, wells (Fig. 2a). After a 24 h incubation period, the plates were photographed individually with a ruler, which served as a reference while measuring zones of inhibition.

Image analysis was done using Adobe Photoshop CS6 (Adobe Systems) and Image J (20). The borders around the zones of inhibition were not always well defined. A photo training process similar to that described by Jorgensen et al. (21) was used to read pixel values and demarcate the zones of inhibition. In analyzing the data, the goal was to detect variations in zone diameters between the extracts used separately and the extracts used in combination. Analysis of Variance (ANOVA) was used to detect if the differences were statistically significant. “Petri Dish ID” was added as a block factor as there could be some variances between each petri dish. This made the study a two-factor ANOVA. “Blocks” and “substance added” were the independent variables and “diameter of zone of inhibition” was the dependent variable. This study is a Mixed Model ANOVA because “petri dish ID” is a random effect. In RStudio (22), using the lme4, lmerTest and lsmeans packages, Type III Analysis of Variance with Satterthwaite's method was performed. This was followed by a *post hoc* Tukey test to compare diameters of the zones of inhibition and detect synergy with a confidence interval of 95%. Each well diffusion assay was repeated three times.

### Checkerboard assay.

Serial dilutions of cranberry extracts, manuka honey and MGO were prepared with 600 μL of sterile water. Corning Falcon polystyrene 96-well microplates with nontreated surfaces (Fisher Scientific, USA) were used in a clean laminar flow hood for the checkerboard assay to check for synergy as described by (14). 100 μL of BHI broth were added to each well of the 96-well plate. 20 μL of S. mutans at 0.5 McFarland standard turbidity were added to each well, except the last column (column 12) of the well. These wells served as the negative growth control. Column 11 containing only media and the bacteria served as a positive growth control. 50 μL of each concentration of cranberry extract was added to wells in separate columns (columns 1 to 8). 50 μL of MGO concentrations were added to rows A to H. This formed a checkerboard pattern with various concentrations of cranberry in the columns, combined with various concentrations of MGO in the rows (Fig. 3a). This 96-well plate was then placed overnight in a 5% (vol/vol) carbon dioxide (CO_2_) incubator at 37°C. C. After 18 to 24 h, the 96-well plate was analyzed for bacterial growth. The lowest wells without any growth in each row and column, adjacent to the wells with bacterial growth form the “growth/no growth interface” (Fig. 3b). The first concentration of a bioactive agent without any bacterial growth is the MIC. If A and B refer to the individual agents, the fractional inhibitory concentration (FIC) index was calculated for each well along the growth/no growth interface using the formula:

FIC index=FIC of A+ FIC of B

where,

FIC of A = MIC of A in combination/MIC of A alone.

FIC of B = MIC of B in combination/MIC of B alone.

The mean FIC is the average of the FICs of each well along the growth/no growth interface. The mean FIC index method (23) was used to interpret the checkerboard assay and determine synergy. If the mean FIC index was over 1, the combination is synergistic. Combinations with mean FICs between 1 to 4 are additive. When the mean FIC is more than 4, the pairs are antagonistic. This methodology was applied to all five cranberry extracts, manuka honey and MGO and studied in combinations. Each checkerboard assay was repeated three times.
